# Global adoption of single-shot targeted intraoperative radiotherapy (TARGIT-IORT) for breast cancer—better for patients, better for healthcare systems

**DOI:** 10.3389/fonc.2022.786515

**Published:** 2022-08-11

**Authors:** Jayant Sharad Vaidya, Uma Jayant Vaidya, Michael Baum, Max Kishor Bulsara, David Joseph, Jeffrey S. Tobias

**Affiliations:** ^1^ Division of Surgery and Interventional Science, University College London, London, United Kingdom; ^2^ Medical Sciences Division Brasenose College, University of Oxford, Oxford, United Kingdom; ^3^ Department of Biostatistics, University of Notre Dame, Fremantle, WA, Australia; ^4^ Department of Radiation Oncology, Sir Charles Gairdner Hospital, Perth, WA, Australia; ^5^ Department of Clinical Oncology, University College London Hospitals, London, United Kingdom

**Keywords:** TARGIT IORT, breast cancer, radiation therapy, lumpectomy (breast conserving surgery), TARGIT, IORT, radiotherapy, partial breast irradiation

## Abstract

**Micro abstract:**

Targeted intraoperative radiotherapy (TARGIT-IORT) is delivered immediately after lumpectomy for breast cancer. We estimated its impact. At least 44,752 patients with breast cancer were treated with TARGIT-IORT in 260 centres in 35 countries, saving >20 million miles of travel and preventing ~2,000 non–breast cancer deaths. The TARGIT-IORT website (https://targit.org.uk/travel) provides maps and tools to find the nearest centre offering TARGIT-IORT and travel savings.

**Background:**

Targeted intraoperative radiotherapy (TARGIT-IORT) delivers radiotherapy targeted to the fresh tumour bed exposed immediately after lumpectomy for breast cancer. TARGIT-A trial found TARGIT-IORT to be as effective as whole-breast radiotherapy, with significantly fewer deaths from non–breast cancer causes. This paper documents its worldwide impact and provides interactive tools for clinicians and patients.

**Method:**

Centres using TARGIT-IORT provided the date of the first case and the total number of patients. We plotted these data on a customised Google Map. An interactive web-based tool provided directions to the closest centre. Using the data from the TARGIT-A trial, we estimated the total savings in travel miles, carbon footprint, and the number of non–breast cancer deaths that might be prevented.

**Results:**

Data from 242 (93%) of the 260 centres treating patients from 35 countries were available. From the first patient treated in 1998 to early 2020, at least 44,752 women with breast cancer have been treated with TARGIT-IORT. The TARGIT-IORT website (https://targit.org.uk/travel) displays the Google Map of centres with number of cases and an interactive tool for patients to find the nearest centre offering TARGIT-IORT and their travel savings. Scaling up to the already treated patients, >20 million miles of travel would have been saved and about 2,000 deaths prevented.

**Conclusion:**

One can ascertain the number of patients treated with a novel treatment. These data show how widely TARGIT-IORT has now been adopted and gives an indication of its beneficial worldwide impact on a large number of women with breast cancer.

## Clinical practice points

- **What is already known about this subject?** Targeted intraoperative radiotherapy (TARGIT-IORT) delivers radiotherapy targeted to the fresh tumour bed exposed immediately after lumpectomy for breast cancer. TARGIT-A trial found TARGIT-IORT to be as effective as whole-breast radiotherapy, with significantly fewer deaths from non–breast cancer causes. This paper documents its worldwide impact and provides interactive tools for clinicians and patients.
- **What are the new findings?** We ascertained that by early 2020, at least 44,752 women with breast cancer have been treated with TARGIT-IORT in 260 centres in 35 countries. We provide at the TARGIT-IORT website (https://targit.org.uk/travel) the Google Map of centres with number of cases and an interactive tool for patients to find the nearest centre offering TARGIT-IORT and their travel savings. We also estimated that, by now, >20 million miles of travel would have been saved and about 2,000 deaths prevented.
- **How might it impact on clinical practice in the foreseeable future?** In addition to hard randomised evidence proving survival and quality of life benefits clinical practice is often prompted by seeing what our peers are doing. Dissemination of these data showing widespread adoption of the technique would further increase awareness and utilisation of this patient-centred approach amongst patients, clinicians, and policymakers.

## Introduction

A large proportion of patients with small breast cancers can be effectively treated by a lumpectomy and radiotherapy, rather than a mastectomy. Radiotherapy is traditionally given to the whole breast.

In the mid-1990s, targeted intraoperative radiotherapy (TARGIT-IORT) (1–3) was proposed as a radical new approach. This treatment delivers effective radiotherapy targeted to the fresh tumour bed exposed immediately after lumpectomy (4, 5) while sparing nearby tissues and nearby vital organs such as the heart and lung.

In pilot studies starting from 2 July 1998, the safety and feasibility of this novel approach combining surgery and radiotherapy were confirmed (1–3), and the TARGIT-A randomised trial was proposed in 1999 (6) comparing risk-adapted single-dose TARGIT-IORT during lumpectomy *vs*. conventional fractionated whole-breast external beam radiotherapy (EBRT) given daily for several weeks (6–8).

Long-term outcomes of the TARGIT-A trial found it to be as effective in terms of breast cancer outcomes and that it led to fewer deaths from other causes (9). Further pre-planned subgroup analysis found that these results are valid for all invasive ductal carcinoma tumour subtypes; there is an overall survival benefit of 4.4% at 12 years in those with grade 1 or 2 tumours (n = 1,797) and identical overall survival in grade 3 cancers (n = 443) (10). Unlike the poor prognosis faced by patients who have a local recurrence after EBRT, those who receive TARGIT-IORT maintain their excellent prognosis even after local recurrence (10). Other benefits included lower radiation related toxicity (11–18), reduced pain, and better quality of life (17, 19–23). When given a choice, TARGIT-IORT is preferred by patients over other methods of radiotherapy or “no-radiotherapy” (24–30). An online tool can guide clinicians in decisions about additional whole-breast radiotherapy after TARGIT-IORT (https://targit.org.uk/addrt) (10).

The adoption of TARGIT-IORT for standard clinical practice has grown considerably over the last 20 years. In this short paper, to assess the worldwide impact of TARGIT-IORT, we aimed to count the number of patients treated with TARGIT-IORT around the world, as well as to estimate the total benefits to the patient, in terms of the saving of travel distance, time, and reduction of transport-related carbon footprint and reduced deaths from other causes.

The TARGIT-A trial was initiated by an academic insight and collaboration with the industry was solely for the development of the device. The study was sponsored by University College London Hospitals (UCLH)/UCL Comprehensive Biomedical Research Centre. Funding was provided by UCLH Charities, National Institute for Health Research (NIHR) Health Technology Assessment programme (HTA 07/60/49), Ninewells Cancer Campaign, National Health and Medical Research Council, and German Federal Ministry of Education and Research (BMBF) FKZ 01ZP0508. The infrastructure of the trial operations office in London, UK, was supported by core funding from Cancer Research Campaign (now Cancer Research UK) when the trial was initiated. In the extended follow-up of the TARGIT-A trial (TARGIT-Ex; funded by the HTA programme of the National Institute for Health Research, Department of Health and Social Care in the UK, HTA 14/49/13), we are continuing the follow up of TARGIT-A trial patients in the UK by direct patient contact and via UK national databases.

We are also currently inviting women who would fall outside the eligibility criteria of the TARGIT-A trial to participate in the TARGIT-B(oost) trial (funded by HTA 10/104/07), already opened in 38 centres internationally, which is comparing TARGIT-IORT as a tumour bed boost with EBRT boost in younger women or women who have higher risk disease to test for superiority in terms of local control and survival.

## Method

Since the first case was performed in London in 1998, an international network has been developed between centres using TARGIT-IORT. Therefore, the contact details of a large proportion of the centres were available. Using Google forms and electronic communication, we requested the date when the first patient with breast cancer was treated with TARGIT-IORT at their centre and how many such patients were treated by their centre in total. We did not restrict this to those centres that had participated in the TARGIT trials. If, after repeated attempts, there was no response from a centre, then we included the name of the centre without the number of cases. We also queried the German National Database (https://www.destatis.de/) using the codes 8.52d, 8-523.6, and 8-521. Such databases were not available for other countries. We collected data until just before the COVID-19 pandemic started. Using Google My Maps, each hospital was displayed on an interactive map, showing the date of the first case and the total number of cases performed at the centre, along with directions to a chosen hospital.

In addition to avoiding the hospital visit required to plan radiotherapy, the large majority of patients (eight out of every 10) who received TARGIT-IORT would avoid 15 to 30 daily trips to the hospital where they would have undergone conventional whole-breast radiotherapy. Therefore, we made an estimate of the total savings by the patient—in terms of travel miles, travel time, and carbon footprint, using the methodology described previously (31). Our previous work (31) had found that patients in the TARGIT-A trial, mostly from urban areas in the UK, saved an average of 305 miles of travel, whereas those in semi-urban areas saved 753 miles. This calculation was based on the total number of hospital trips the patients saved when they were randomised to the TARGIT-IORT arm compared with the EBRT arm in the randomised TARGIT-A trial. The distance travelled for each trip was individually calculated by inputting in Google Maps API, the addresses of the patient, and the treating hospital where the EBRT was given. The total miles saved were used to calculate the amount of CO_2_ saved using standard emissions for a medium sized car. This estimate takes into account the additional travel required in the 20% of patients who are recommended whole-breast EBRT. It has been estimated that 55% of the world population lived in urban areas in 2018 (32). In this paper, we used the UK figures for travel savings and assumed a larger proportion of patients (66% rather than 55%) will be urban dwellers. We prepared an interactive web application to make individual estimates. These tools were tested by patients, and their feedback was used for making improvements.

Long-term results of the TARGIT-A trial (9) (**Supplementary Figure 1**) found no difference any breast cancer outcome or breast cancer–specific mortality but a significant reduction in non–breast cancer mortality (HR, 0.59; 95% CI, 0.40 to 0.86; P = 0.005) such that it was 5.41% for TARGIT-IORT and 9.85% for EBRT. The difference was 4.44% (95% CI of the difference being 2.5% to 6.4%). This estimate is consistent with that of overall survival improvement in patients with grade 1 and grade 2 cancers that formed a large subgroup of patients in the trial contributing 1,796 out of the total of 2,298. In a pre-specified subgroup analysis (with its usual caveats), overall survival was significantly better in this subgroup by 4.4% (HR, 0.72; p = 0.0361). We used this absolute difference in deaths, i.e., 4.4 fewer deaths over 12 years per 100 patients treated, to estimate the global impact of using TARGIT-IORT in terms of mortality reduction amongst patients already treated around the world.

We used STATA 16 for statistical analysis.

## Results

Data from 242 (93%) of the 260 centres were available. Data from 31 of 64 centres (n = 8021) in Germany were available directly from investigators and the remaining 33 (n = 8044) from the German national database. Of these 260 centres, 33 had participated in the TARGIT-A trial.

The first patient with breast cancer was treated with TARGIT-IORT on 2 July 1998 at the Middlesex hospital (now part of University College London Hospitals), University College London. Since then, we found that TARGIT-IORT has been used in 35 countries, and at least 44,752 patients with breast cancer have been treated (**Table 1**). The total numbers of patients known to have been treated are approximately 30,000 in Europe, 9,000 in North America, 3,000 in Asia Pacific, 2,000 in South/Central America, 500 in the Middle East, and 200 in Africa. **Table 2** has the list the collaborating centres.

**Table 1:** **a**) Number of centres that have treated patients with breast cancer with TARGIT-IORT around the world and **b**) number of patients treated in each of the world regions.

a) Number of centres per country and region			
Region	Country	Number of centres	Centres from where the number of patients is available
Africa	South Africa	1	1
**Africa Total**		**1**	**1**
Asia and Pacific	Australia	3	3
	China	13	13
	India	2	2
	Malaysia	4	4
	New Zealand	1	1
	Philippines	1	1
	Singapore	1	1
	South Korea	1	1
	Thailand	1	1
	Vietnam	1	0
**Asia and Pacific Total**		**28**	**27**
Europe	Austria	1	1
	Belgium	1	1
	Bulgaria	1	1
	Denmark	1	1
	France	12	12
	Georgia	1	1
	Germany	63	65
	Israel	9	9
	Italy	5	5
	Norway	1	1
	Poland	8	2
	Russia	12	3
	Spain	3	3
	Switzerland	6	6
	Turkey	4	2
	United Kingdom	11	11
**Europe Total**		**140**	**124**
Middle East	Iran	2	2
	Saudi Arabia	3	3
**Middle East Total**		**5**	**5**
North America	Canada	2	2
	USA	72	71
**North America Total**		**74**	**73**
South/Central America	Brazil	4	4
	Mexico	3	3
	Peru	2	2
	Venezuela	3	3
**South/Central America Total**		**12**	**12**
**Grand Total**		**260**	**242**
b) Number of patients treated per region			
Region			Number of patients treated
Africa			179
Asia pacific			2,803
Europe			29,716
Middle East			1,009
North America			9,019
South America			2,026
**Total**			**44,752**

Since the initial submission, there is another country to this list: Argentina.

**Table 2:** TARGIT-IORT global collaborators.

Centre	Contributors
University College London Hospital, London, UK	Jayant S. Vaidya, Max Bulsara, Michael Baum, Jeffrey S. Tobias, Chris Brew-Graves, Ingrid Potyka, Nick Roberts, Norman Williams
Sir Charles Gairdner Hospital, Perth, WA, Australia	Christobel Saunders, Tammy Corica, David Joseph
University Medical Center Mannheim, Medical Faculty Mannheim, Heidelberg University, Germany	Elena Sperk, Marc Sutterlin, Frederik Wenz
Centro di Riferimento Oncologico, Aviano, Italy	Samuele Massarut, Lorenzo Vinante
Peter Mac Centre, Melbourne, Australia	Boon Chua
Ninewells Hospital, Dundee, Scotland, UK	Douglas Brown, Julie Lindsay
Klinikum der Johann-Wolfgang Goethe-Universität, Frankfurt 60596, Germany	Claus Rödel, Manfred Kaufmann
UCSF Helen Diller Family Comprehensive Cancer Center, San Francisco, CA, USA	Michael Alvarado, Jane Wei
Technical University Munich and Red Cross Munich, Germany	Steffi Pigorsch, Christian Diehl
University of Southern California, USC, USA	Dennis Holmes
Department of Surgical Oncology, Medical University of Lublin, Lublin, Poland	Wojciech Polkowski
Ospedale San Giuseppe di Empoli, Viale Giovanni Boccaccio, 16, 50053 Empoli FI, Italy	Claudio Caponi
Sankt Gertrauden-Krankenhaus, and The Charité – Universitätsmedizin Berlin, Berlin, Germany	Jens Blohmer, Volker Budach, Dirk Böhmer
Ludwig Maximilian University of Munich, Munich, Germany	Montserrat Pazos, Claus Belka, Nadia Harbeck
Herlev Hospital, Copenhagen, Denmark	Henrik Flyger
Princess Margaret Cancer Center, Toronto, Canada	David McCready, Jaime Escallon
Royal Hampshire County Hospital, Winchester, UK	Siobhan Laws, Dick Rainsbury, Ajay Raj
Radiotherapie Hirslanden, Brust-Zentrum Seefeld, Zurich, Switzerland	Gunther Gruber, Barbara Papassotiropoulos, Christoph Tausch
Lafayette Surgical Clinic, 1345 Unity Pl #235, Lafayette, IN 47905, USA	Thomas Summer
Royal Free Hospital, Hampstead and Whittington Hospital, London, UK	Tim Davidson, Mohammed Keshtgar, Jayant S. Vaidya, Katharine Piggott
Sentara Surgery Specialists, Hampton, USA	Richard Hoefer, Song Kang
Saarland University Medical Center, Homburg, Germany	Marcus Niewald
University Hospital of Zurich, Switzerland	Konstantin Dedes
University of Science and Technology (NTNU) Trondheim, Norway	Steinar Lundgren
University of Nebraska Medical Center, Buffet Cancer Center, S 42nd St and Emile St, Omaha, NE 68198, USA	Deborah Spence, James Edney
Guy’s Hospital, London, UK (now at Oxford University Hospital)	Michael Douek, Joyce Akinwale
Ashikari Breast Center, St. Johns Riverside, Dobbs Ferry, NY, USA	Pond Kelemen, Andrew Ashikari
Vassar Brothers Medical Center, Poughkeepsie, NY, USA	Daniel Lackaye, Dan Pavord, William Rausch, Dimitrios Papadopoulous, Camilo Torres
Institute de Cancerologie de l’Ouest René Gauducheau, Nante, France	Magali Le-Blanc-Onfroy
Medical School Hannover, Germany	Michael Bremer, Park-Simon, Tjoung-Won
Instituto Oncologico Veneto, Padoa, Italy	Fernando Bozza, Franco Berti, Silvia Michieletto
Institut Bergonié, Bordeaux France	Beatrice Gonzalves, Christel Breton-callu, Adeline Petit
St John and St Elizabeth Hospital, London, UK	Mohammed Keshtgar
Whittington Hospital, London, UK	Jayant S. Vaidya, Jeffrey S. Tobias
CHU Morvan, Brest, France	Pierre Francoise Dupre, Pradier Olivier, Chajara Abdesslam, Sarah Quillevéré
Beverley Hill Cancer Centre (Helen Rey), CA 90210, USA	Dennis Holmes
Imam Abdulrahman Bin Faisal University, Dammam, Kingdom of Saudi Arabia	Maha Abdel Hadi
Centre Léon Bérard, 28 Prom. Léa and Napoléon Bullukian, 69008 Lyon, France	Severine Racadot, Jean-Noel Badel
Princess Grace Hospital, London, UK	Jayant S. Vaidya, Jeffrey S. Tobias
Center Georges-François Leclerc - Dijon, France	Etienne Martin, Charles Coutant, Karine Peignaux-Casasnovas, Magali Rouffiac, Gilles Truc, Fabienne Bidault, Mathieu Gonod
Memorial University Medical Center, Savannah, GA, USA	Aaron Pederson, William Burak
Universidad Fernando Pessoa Canarias. Hospital de Gran Canaria Dr Negrín, Gran Canaria, Spain	Pedro Lara, Beatriz Pinar Sedeño
CLEVELAND CLINIC FOUNDATION, Cleveland, OH, USA	Stephanie Valente, Sheen Cherian, Stephen Grobmyer
Princess Alexandra Hospital, Harlow, UK	Julian Singer, Ashraf Patel, Veronica Grassi, Bijan Ansarimohabadian
Gangnam Severance Hospital, Yonsei University, Seoul	Joon Jeong
Aurora Baycare Medical Centre, Green Bay, WI, USA	William Owens
Institut Universitaire du Cancer de Toulouse Oncopole, Centre Claudius Regaud, Toulouse, France	Izar Francoise
Institut Catalan de Oncología. Hospital de bellvitge, Hospital Duran i Reynals, Avinguda de la Gran *Via* de l’Hospitalet, 199-203, 08908 L’Hospitalet de Llobregat, Barcelona, Spain	Ferran Gueda, Arancha Eraso, Evelyn Martinez, Maria Laplana, Maria Jesus Pla, Pablo Saldaña, Roberto Martín Vaello
Great Western Hospital, Swindon, UK	Nathan Coombs, Shiroma DeSliva Minor, David Dommett
Morgantown, Health Sciences Centre, WV, USA	Geraldine Jacobson
Centre Hospitalier Universitaire (APHM CHU Nord and Hopital de la Timone), Marseille, France	Didier Cowen, Jean Baptiste Haumonte, Aubert Agostini, Corinne Aquaron, Natacha Nomikossoff
Beijing Cancer Hospital(2), No. 52 Fucheng Road, Haidian District, Beijing (Ding Hui Temple), China	Xinguang Wang, Chang Cheng
University Malaya Medical Centre (UMMC), Kuala Lumpur, Malaysia	Nur Aishah Mohd Taib, See Mee Hoong, Suniza Jamaris, Teh Mei Sze, Teoh Li Ying, Marniza Saad, Anita Zarina Bustam, Rozita Abdul Malik, Nur Fadhlina Abdul Satar
Centre François Bâclasse, Caen, Normandy, France	Serge S. Danhier, Julien Geffrelot, Alain Batalla, Jean Francoise Le Brun, Sandrine Martin-Francoise, Helen Planque
William Beaumont Hospital, Detroit, MI, USA	Nayana Dekhne, Peter Chen, Blerina Pople
Lakeland Health, St Joseph, MI, USA	Benjamin T. Gielda
Queen Sirikit Centre for Breast Cancer, King Chulalongkorn Memorial Hospital, Bangkok, Thailand	Kris Chatamara, Adhisabandh Chulakadabba, Sikrit Denariyakoon
Gauteng, Netcare Milpark Hospital, South Africa	Carol Benn, Yastira Ramdas
Rest of German centres (not all are listed) have treated a total of 7,853 patients with breast cancer	
New York Medical College, NY, USA	Basil Hilaris
Maria Skłodowska-Curie Memorial Cancer Centre and Institute of Oncology (MSCNRIO) Gliwice branch, Gliwice, Poland	Jerzy Wydmański, Żaneta Kaniszewska-Dorsz, Andrzej Tukiendorf
Summit Hospital (Oncologics), Baton Rouge, LA, USA	John Head, Bob Elliot
Carmel Medical Center, Haifa, Israel	Mariana Steiner
Klinikum Augsburg, University Medical Center Augsburg, Germany	Henning Kahl
Casa di Cura Quisisana, Rome, Italy	Stefano Drago
University of Regensburg Radiotherapy, Caritas - Krankenhaus St. Josef’, Germany	Oliver Kölbl
Klinik Hirslanden, Spital Männedorf, Männedorf, Switzerland	Gunther Gruber, Barbara Papassotiropoulos, Christoph Tausch
Mammazentrum, Krankenhaus Jerusalem, Moorkamp 2-6, Hamburg 20357, Germany	Florian Würschmidt (Radiologische Allianz Hamburg), Kay Friedrichs
Diakonie Klinikum Hamburg, Hamburg 20259, Germany	Florian Würschmidt (Radiologische Allianz Hamburg), Christoph Lindner
Renaissance Surgical Memorial Care Pacific Breast Care Center, Costa Mesa, CA, USA	Alice Police
Klinikum St. Marien Amberg, Amberg 92224, Germany	Hipp Matthias, Klaus Graaf, Tanja Eberl, Thomas Papathemelis, Tanja Hauzenberger, Anton Scharl
Klinikum Nürnberg Nord, Klinik für Frauenheilkunde und Geburtshilfe Universitätsklinik der Paracelsus Medizinischen Privatuniversität	Cosima Brucker
Indo-American Cancer Institute, Hyderabad, India	Sushila Narayan, Mohan Vamsy
Oregon Health Science University, Portland, OR, USA	Susha Pillai, Arpana Naik
University of Florida, Gainesville, FL, USA	Lisa Spiguel, Paul Okunieff, Natalie A Lockney, Jian Wu, Chihray Liu
Institute for Breast Diseases, Fucam Hospital, Mexico City, Mexico	Antonio Maffuz-Azis, Sergio Rodrigez-Cuevas, Judith Huerta-Bahena, Carlos Alberto Dominguez-Reyes, Jorge Anselmo Vazquez-Reyes
Marienhospital Bottrop, Josef-Albers-Straße 70, 46236 Bottrop, Germany	Hans-Christian Kolberg
University of Cologne, Faculty of Medicine and University Hospital of Cologne, Germany	Wolfram Malter, Stefan Krämer, Peter Mallmann, Karolina Jablonska, Wolfgang Baus, Simone Marnitz
Trinity Medical Center, Birmingham, AL, USA	William Thompson
California Pacific Medical Center, San Francisco, CA, USA	John Lee, Terry Pierce
Vorarlberger Krankenhaus- betriebsges.mbH, Carinagasse 47, A-6807 Feldkirch, Austria	Rita Alton
Northern Westchester Hospital, Mount Kisco, NY, USA	Stephen Iorio
Klinikum Westfalen, Am Knappschaftskrankenhaus 1, 44309 Dortmund, Germany	Mohammed Yossof Karim-Payab, Heidemarie Tonscheidt Head, Frank Schmolling
King Abdulaziz University Hospital, Jeddha, Saudi Arabia	Yasir Bahadur
Northwestern University Hospital, 251 E Huron St, Chicago, IL 60611, USA	Eric Donnelly, Hualin Zhang
Moffitt Cancer Center, Tampa, FL, USA	Christine Laronga
Marien Hospital and St Barbara Klinik, Hamm Heessen GmbH	Jany Ralf, Hermann Wiebringhaus, Frank Holms, Thilo Vormann, Tobias Tan-Tjen, Norbert Lang
Kreiskrankenhaus Gummersbach, Klinik für Strahlentherapie, Wilhelm Breckow Allee 20, 51643 Gummersbach, Germany	Peter Vacha, Golamabu Zakaria, Magdolna Bajnok, Anja Weishap
Raheja Hospital, Mumbai, India	Sanjay Sharma
Klinikum Stuttgart - Katharinen Hospital, Germany	M. Münter, U. Köppen, N. Wegner, J. Schuster, A. Golle, S. Baumbach, S. Staubus, U. Karck
Klinikum St. Georg GmbH, Saxony, Leipzig, Germany	André Liebemann, Marion Hindemith, Susanne Miethe, Niels-Karsten Bär, Cornelius Walter, Uwe Köhler
Institut Regional du Cancer de Montpellier- ICM Val d’Aurelle, Montpellier, France	Claire Lemanski, David Azria, Marian Gutowski
Bay Area Cancer Physicians at Summit Medical Center, Oakland, CA, USA	Valery Uhl
Sutter Medical Center, Sacramento, USA	Jeannine Graves
Städtisches Klinikum Lüneburg, Lueneburg, Germany	Stefan Dinges, Eric Boetel
Brustzentrum Rhein-Kreis-Neuss, Johanna-Etienne-Krankenhaus Neuss, Germany	Georg Unruh, Susanne Coslar
Cornell University, New York, NY, USA	Alex Swistel, Samuel Trichter, John Ng
Hôpitaux Universitaire de Genève, Geneva, Switzerland	Pelagia Tsoutsou, Vincent Van Hung, Odile Fargier Bochaton, Thanh Giang Lam
Institut Paoli Calmettes, Marseille, France	Agnes Tallet, Gilles Houvenaeghel, Monique Cohen, Leonel Varela-Cagetti, Laurence Gonzague, Véronique Favrel, Marguerite Tyran, Pierre Annède, Eric Lambaudie, Sandrine Rua, Max Buttarrelli
Advocate Good Shepherd Hosp, Barrington, 1301 S Barrington Rd, Barrington, IL, USA	Barry Rosen, Brian Tom
Community Surgery Center North, 1550 East County Line Road, Indianapolis, IN 46227, USA	Susan Chace Lottich, Darrel Ross
University of Iowa Hospitals and Clinics, Iowa City, IA, USA	Timothy Waldron, Wenqung Sun, Allison W Lorenzen
Ammerlandklinik Westerstede, Germany	Robert M. Hermann
National Cancer Centre, 11 Hospital Drive, Chow, Singapore	Kong Wee Ong, Veronique K.M. Tan, Fuh Yong Wong, Eu Tiong Chua, Richard M.C. Yeo, Sue Zann Lim
Riyadh Military Hospital, Riyadh, Saudi Arabien	Esam Murshid, Marouf Adili
St. Louis Hospital, APHP, Paris, France	Christophe Hennequin
Specialist Center for Radiation Therapy and Laboratory Medicine, Steinbacher Hohl 2-26, 60488 Frankfurt am Main, Germany	Uta Kraus-Tiefenbacher, Volker Möbus
Littleton Adventist Hospital, Littleton, CO, USA	Darlene Bugoci, Ellen Buchannan, Jodi Widner, Justin Keener
The Hoffberger Breast Center at Mercy, 227 St Paul Pl, Baltimore, MD 21202, USA	Neil B. Friedman
Holy Cross Hospital, Ford Lauderdale, FL, USA	Omar Rashid, Joseph J Casey, Marnie Kaplan, Lav Goyal, Irina Frosman
OLV Hospital Aalst, Moorselbaan 164, 9300 Aalst, Belgium	Adelheid Roelstraete, Koen Traen
Washington Hospital Center, Washington, D.C., USA	Eleni A Tousimis, Marc Boisvoir
Kantonsspital Münsterlingen und Frauenfeld, Spital Thurgau AG, Switzerland	Hans Reichardt, Christiane Reuter
Military Region General Hospital of Lanzhou, No. 333, South Binhe Road, Qilihe District, Lanzhou City, China	Zhao Qingli
Lindenhofgruppe Engeriedspital, Bern, Switzerland	Armin Thoeni, Gilles Berclaz, Jacqueline Vock, Karin Muench
St. Thomas Ascension Midtown Hospital, (previously Baptist Hospital), Nashville, TN, USA	Pat Whitworth, Kenneth Lloyd, Julian Heitz
Academician F. Todua Medical Center- Research Institute of Clinical Medicine, Tbilisi, Georgia	Mikheil Janjalia, Irakli Sixarulidze, Natalia Jankarashvili, Maia Topeshashvili, Mikheil Kavtaradze
The First Affiliated Hospital of Guangzhou Medical University, No. 151, Yanjiang West Road, Yuexiu district, Guangzhou, China	Wenbo Zheng
Instituto Nacional De Cancerologia (INCAN), Mexico City, Mexico	Enrique Bargallo, Christian Flores, Gabriel Santiago
MedStar Georgetown University Hospital, 3800 Reservoir Rd NW, Washington, DC 20007, USA	Eleni Tousimis
Guangdong Provincial People’s Hospital, No. 106 Zhongshan 2nd Road, Guangzhou City, Guangdong Province, China	Yi Pan, Wei Huang
Hudson Valley Hospital Center, Cortland Manor, NY, USA	Pond Keleman
Franziskushospital Harderber, Radiologische Klinik Alte Rothenfelder Landstrasse 23 D-49124 Georgsmarienhütte, Germany	Otfried Sauer, Albert von der Assen
St.Luke’s Hospital Anderson Campus, Easton, PA, USA	Lee Riley
Cancer Treatment Centers of America at Southeastern, Newnan, GA, USA	Anita Johnson, John Swanson, Christian Hyde, Joseph Dick, Patricia Young
Cancer Treatment Centers of America @ Western Regional Medical Center, Goodyear, AZ, USA	Simon Lam, Matt West
The First Pavlov State Medical University of St. Petersburg, Academition Pavlov Str.9, St. Petersburg, Russia	Alexey G. Manihas, Babeshkin Roman Nikolaevich
American British Cowdray (ABC) Medical Center, Mexico City, Mexico	Jorge Omar Hernandez Oviedo, Dolores De La Mata, Jose Hinojoso, Fabiola Flores, Carlos Robles, Bargallo Enrique, Antonio Maffuz-Azis
Marietta Memorial Hospital, Marietta, OH, USA	Teressa Valentine, Rajendra Bhati, Srini Vasan
Focus Radiotherapy, 209 Shakespeare Rd, Milford, Auckland, New Zealand	Erica Whineray Kelly
Columbia University Medical Center, New York, NY, USA	Eileen Connolly, Sheldon Feldman, Bret Taback
Clinica Leopoldo Aguerrevere, Caracas 1080, Miranda, Venezuela	Alecia Cosson, Ricardo Paredes, Gerardo Hernandez, Juan Rasquin, Adriana Pesci, Francisco Dona, Elizabeth González
John Muir Health Care, Walnut Creek, CA, USA	William Bice, Marjaneh Moini, Suzanne Clements
Moscow City Hospital №. 57, Moscow, Russia	Dmitry Bondar
McGill University Health Center, 1001 Decarie Blvd, Montreal, Quebec H4A 3J1, Canada	Marija Popovic, Bassam Abdulkarim, Peter Watson, Jan Seuntjens
Loyola University Medical Center, Maywood, IL, USA	William Small Jr., T. Refaat, T. Thomas, C. Hentz, S. Gros
North Shore Long Island Jewish, Health System Center for Advanced Medicine, 450 Lakeville Road, Lake Success NY 11042, USA	Lin Wang
Lenox Hill Hospital, New York, NY, USA	Alice Police
Diagnosticos C.A., Barcelona, Estado Anzoategui, Venezuela	Eduardo Benavides, Ivan Gonzalez
Instituto Imor, Instituto Médico de Onco-Radioterapia. Carrer de les Escoles Pies, 81, 08017 Barcelona, Spain	Benjamin Guix, Iván García, Manel Algara, Miquel Puig
Lahey Hospital and Medical Center, 41 Burlington Mall Road, Burlington, MA 01805, USA	Per Halvorsen, Andrea McKee
Meir Medical Center, Israel	Bella Nisenbaum
Medipol University, Istambul, Turkey	Hale Basak Caglar, Dilek Unal
Kaplan Medical Center, Rehovot, Israel	Tanir M Allweis
Hospital Sao Rafael, Salvador, Brazil	Arthur Rosa, Ezio Novais Dias
Kaiser Oakland Medical Center, Oakland, CA, USA	Veronica Shim
Cancer Research Center, Shohada Tajrish Hospital, Shahid Beheshti University of Medical Sciences, Tehran, Iran	Mohammad Esmail Akbari
Instituto Nacional de Enfermedades Neoplásicas, Suquilo, Lima (INEN), Peru	Gustavo Sarria, Jose Antonio Galarreta, Julio Abugattas
Ha’emek Medical Center, Afula, Israel	Hershko Da
Lee Health Regional Cancer Centre, Fort Myers, FL, USA	David Rock, Alan Brown Jr.
Krankenhaus Weinheim, Gesundheitszentren Rhein-Neckar GmbH, Germany	Lelia Bauer, Bettina Müller
Universitätsklinikum Bonn, Germany	Frank Giordano, Stephan Garbe, Christopher Schmeel
University of California Irvine Medical Center, Orange, USA	Alice Police, Erin Lin, Jeffery Kuo
Assuta Medical Centers, HaBarzel St 20, Tel Aviv-Yafo, Israel	Daphne Levin, Yonina Tova, Vladislav Greenberg
Beilinson/Rabin Medical Center, Petah Tikva, Israel	Eran Sharon
The First Affiliated Hospital of Zhengzhou University, No. 1 Jianshe Dong Road, Zhongyuan District, Zhengzhou City, Henan Province, China	Li Guowen
University of California Los Angeles (UCLA), Medical Center Harbor, Torrance, USA	Christine Dauphine, Junko Ozao-Choy, Chad Sila, Eric Frank, Katherine Magat
Soroka Medical Center, Beer Sheba, Israel	Ravit Agassi
Bethesda North Hospital, OH, USA	Jessica Guarnaschelli, Ching Ho, Peter Sandwall
Helios Klinikum Bad Saarow, Germany	Stephan Koswig, Gerlinda Kho, Marén Sawatzki, Justyna Polowy
Inova Fairfax Hospital, Falls Church, VA, USA	Stella Hetelekidis, Lonika Majithia, Ashish Chawla, Michael Eblan, Sara Bruce, David Weintritt, Constanza Cocilovo, Robert Cohen, Kirsten Edmiston
Hospital Alemão Oswaldo Cruz, São Paulo, Brazil	Rodrigo Hanriot, Patricia B. Aguilar, Douglas G. Castro, Guilherme RM Gondim
The First Affiliated Hospital, Sun Yat-sen University, No. 58, Zhongshan Second Road, Yuexiu District, Guangzhou, China	Ying Lin
Emory University Midtown Hospital, Atlanta, GA, USA	Rogsbert Phillips, Karen Godette
Ospedale dell’Angelo - Mestre VENEZIA, *Via* Paccagnella, 11, 30174 Venice VE, Italy	Sonia Reccanello
Medicana International Ankara Hospital, Cankaya/Ankara, Turkey	Kaan Oysul
The Second Affiliated Hospital, Sun Yat-sen University(2), No. 107 West Yanjiang road, Guangzhou, Guangdong, China	Lin, Huang, Shi Juntian
The London Clinic, 20 Devonshire Avenue, London, UK	Gerald Gui, Jeffrey S. Tobias, Jayant S. Vaidya, Tim Davidson, Susan Cleator, Simon Stevens
RF Magadan Regional Oncology Centre	Roman Shumel
Newport Beach Surgery Center, CA, USA	Alice Police
Haerbin Medical University Cancer Hospital, No. 150 Haping Road, Nangang District, Harbin City, Heilongjiang Province, China	Zhao Chunbo
Greenwich Hospital, Greenwich, USA	Barbara Ward, Sana Quirk
University Hospital “Tzaritza Joanna – ISUL”, Medical University of Sofia, Bulgaria	Theophil Sedloev, Slavyana Usheva, Iliya Gabrovski, Ivan Terziev
Clinica AUNA Oncosalud, Lima, Peru	Gustavo Sarria, David Martinez
Inova Alexandria Hospital, Alexandria, VA, USA	David Weintritt, Sara Bruce, Tobias Chapman, Lonika Majithia
Fundação Antonio Prudente - Hospital AC Camargo Cancer Center, Sao Paolo, Brazil	Antonio Cassio De Assis Pellizzon, Fabiana Makdissi, Ricardo Fogarolli, Juan Bautista Donoso Collins, Guilherme Rocha Gondim
University of Würzburg, Würzburg, Germany	Bülent Polat, Achim Wöckel, Marcus Zimmermann
California Hospital Medical Center, Los Angeles, CA, USA	Dennis Holmes
Mount Carmel Hospital, Columbus, OH, USA	Shilpa Padia, Malouan Rajagopolan
Sha’arei Zedek Medical Center	Carmon Moshe
Pastornow Cancer Research Center, and Medical Physics Research Center, Mashhad University of Medical Sciences, Mashhad, Iran	Hamid Gholamhosseinian, Roham Salek, Mohammad Naser Forghani, Mahboobeh Sadeghi ivari, Fatemeh Homaei, Kazem Anvari, Gholamhossein Noferesti, Amir Aledavood,
Clinique du Sein, Centre Republique, 99 avenue de la République, 63100 Clermont-Ferrand, France	Christophe Scherer, Doridot Virgenie
The Second Affiliated Hospital of Guangzhou Medical University, 250 Changgang Middle Rd, Haizhu, Guangzhou, Guangdong, China	Hu Xiaowu, Yong He
HELIOS Medical Center Krefeld, Germany	Stefan Krämer, Michael Friedrich, Michael Daum-Marzian, Dilek Saylan, Maike Sellinger
Helios University Hospital Wuppertal, University Witten/Herdecke, Germany	Marc D Piroth, Vesna Bjelic-Radisic, Markus Fleisch, Steffi Marzotko, Bianca Böning, Arnd Röser
The First Hospital Affiliated To AMU(Southwest Hospital), Lihui road, Beibei district, Chongqing, China	Yi. Zhang
Hospital Dr Domingos Luciani, Caracas 1073, Miranda, Venezuela	Carlos Nunez, Berta Prato
Wellington Regional Medical Center, Wellington, FL, USA	Kathleen Minnick, Kishore Dass, Andrew J. Shapiro
Sunway Medical Centre, 5, Jalan Lagoon Selatan, Bandar Sunway, 47500 Petaling Jaya, Selangor, Malaysia	Char Hong Ng
Inova Fair Oaks, 3600 Joseph Siewick Dr, Fairfax, VA 22033, USA	Stella Hetelekidis, Ashish Chawla, Michael Taylor, H Vargas, Moonseong Oh, Kirsten Edmiston
Halifax Hospital, Daytona Beach, FL USA	Domenico Dellicarpini
Advocate Masonic Hospital, Chicago, IL, USA	Barry Rosen
New Mexico Cancer Care Alliance, Albuquerque, New Mexico	Calvin Ridgway
Sun Yat-sen University Cancer Center, No. 651 East Dongfeng road, Yuexiu District, Guangzhou, Guangdong, China	A Long Chen
Subang Jaya Medical Centre, No. 1, Jalan SS12/1A, Ss 12, 47500 Subang Jaya, Selangor, Malaysia	Yip Cheng-Har
Assuta Medical Centre, Haifa, Israel	Abdah-Bortnyak Roxolyana, Rafi Klein
Phelps Hospital, Sleepy Hollow, NY, USA	Alice Police
University of Miami/Jackson Memorial Hospital, Miami, FL, USA	Eli Avisar, Cristiane Takita
Montifiore Hospital, New York, NY, USA	Sheldon Feldman
Rochester Regional Health, 100 Kings Highway South Rochester, NY 14617, USA	Lori Medeiros, Deore Shivaji, Michelle Beaty, Xunyi Xu, Mubin Shaikh, Adi Robinson, Joel Yellin, Meri Atanas
Mount Sinai Hospital, 1468 Madison Ave, New York, NY 10029, USA	Sheryl Green
Hainan Cancer Hospital, No 6, Changbin West 4th St, Xiuying district, Haikou City, Hainan Province, China	Haonan Ran
No. 12 Jiankang Rd, Changan District, Shijiazhuang City, Hebei Province, China	Zhang Ruohui
IMO- Instituto de Mastologia e Oncologia - Goiania - GO - Brazil	Nilceana Maya Aires Freitas, Ruffo Freitas Junior, Alexandre Marchiori, Jean Teixeira Paiva, Lais Tomaz Maya
Legacy Health, Portland, OR, USA	Mark Schray, Nathalie Johnson, Cynthia Aks
Prince Court Medical Centre, 39, Jalan Kia Peng, Kuala Lumpur, 50450 Kuala Lumpur, Wilayah Persekutuan Kuala Lumpur, Malaysia	Harjit Kaur Perdamen
The Medical City, Ortigas Ave, Pasig, Metro Manila, Philippines	Aldine Astrid Arive Basa
Inova Loudoun Hospital, Leesburg, VA, USA	Virginia Chiantella, Lonika Majithia
St John of God Hospital, Subiaco, Perth, Australia	Christobel Saunders
University of Kansas Medical Center (KUMC), Overland Park, Kansas, KS, USA (centre started after the initial submission so numbers not included in the total)	Kelsey Larson, James Coster
Hospital Italiano de Buenos Aires, MEVATERAPIA, Argentina (centre started after the initial submission so numbers not included in the total)	Carola Allemand, Federico Diaz
Fortis Hospital, Bangalore, India (centre started after the initial submission so numbers not included in the total)	Sandeep P. Nayak, Nisha Vishnu

Jayant S. Vaidya, Uma J. Vaidya, Michael Baum, Max Bulsara, David Joseph, and Jeffrey S. Tobias, on behalf of the TARGIT-IORT Global Collaborators. The centres are listed in order when the first case was treated firstly within TARGIT-A trial, then TARGIT-B trial, and then those outside these two trials. This table is not an exhaustive list and includes only those given by contributors who have responded to our emails for collaboration. We apologise for the omission of any names. Up to date list at https://targit.org.uk/travel.

**Figure 1:**
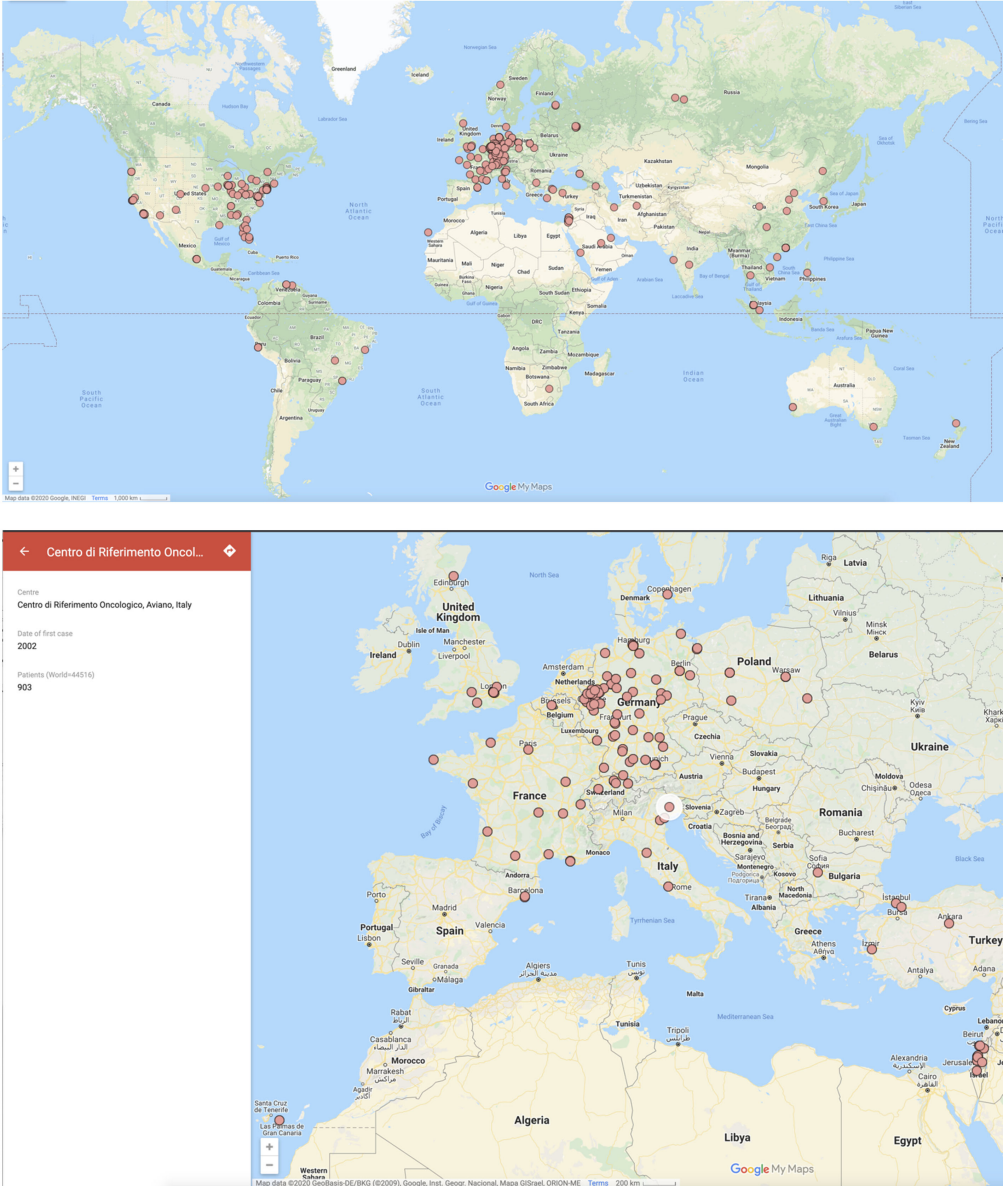
Screenshots of the map of the world with each dot representing a centre that has treated breast cancer with TARGIT-IORT. The name of the centre and the number of cases treated by the centre (if available) are seen in the left-hand pane when you click on the centre in 1b below (the map can be zoomed in). This map is interactive and available at https://targit.org.uk/travel.

**Figure 1** is the screenshot of an interactive Google Map that shows the centres that have offered TARGIT-IORT for breast cancer, the year of their first case, and the number of cases performed as of August 2020. Once the reader clicks on a particular centre, they can get directions to the centre by clicking on the direction arrow on top left corner, next to the name of the centre. The interactive map in **Supplementary Figure 2** shows the number of centres in each country. **Supplementary Figure 3** shows how they have increased since 1998.

Scaling up the journeys saved by avoiding EBRT, because of the use of TARGIT-IORT, to the 44,752 patients, we estimate that over 20 million (20,134,909) miles of travel have already been saved, representing a carbon footprint reduction of 5.6 million kg of CO_2_ emissions.

**Figure 2:**
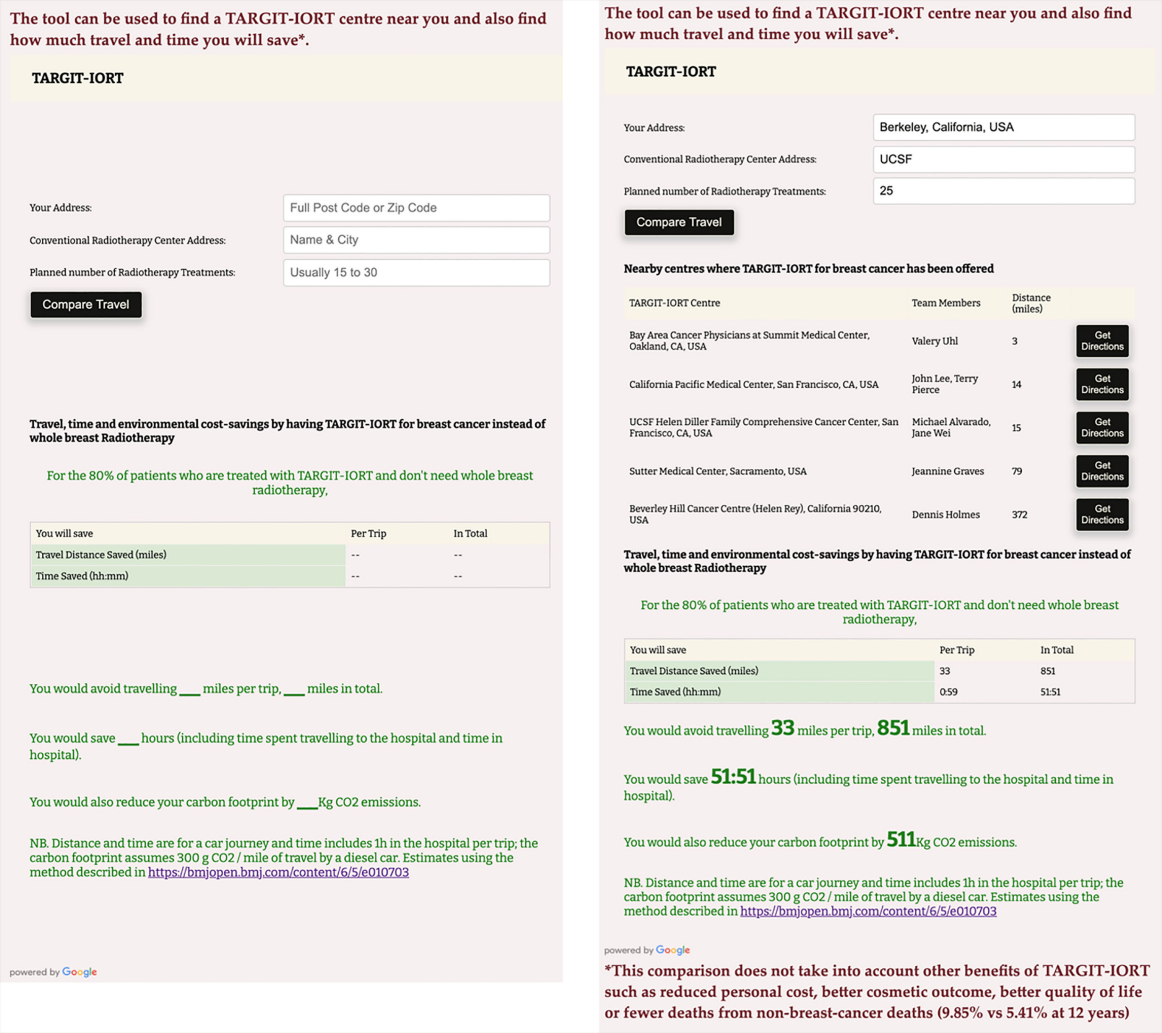
A screenshot of the interactive tool to assess how much an individual patient would save by using TARGIT-IORT in terms of travel distance, time, and carbon footprint. This example is for someone living in Berkeley, California, USA, for example, and going for radiotherapy at the University of Califoria San Francisco UCSF hospital, the closest radiotherapy centre from this house. This interative tool can be accessed at https://targit.org.uk/travel.

**Figure 2** is the screenshot of the interactive tool with which one can find the centre offering TARGIT-IORT closest to one’s home. It will also estimate how much an individual patient would save by using TARGIT-IORT in terms of travel distance, time, and carbon footprint.

These interactive maps and tools can be accessed at https://targit.org.uk/travel.

Scaling up the 4.44% (95% CI, 2.5% to 6.4%) reduction in non–breast cancer mortality to the 44,752 patients treated to date (mid-2020), we estimate that 1,987 (95% CI, 1,129 to 2,845) non–breast cancer deaths from causes other than breast cancer such as cardiovascular and lung problems and other cancers would be prevented.

## Discussion

This paper describes the worldwide adoption of TARGIT-IORT for treatment of early breast cancer over the past two decades. We could confirm that TARGIT-IORT has been used in 260 centres in 35 countries and about 45,000 patients in six continents have been treated. In the process, an estimated 20 million miles of journeys were avoided. Applying the reduction in non–breast cancer mortality, found in the TARGIT-A trial, to the patients already treated around the world suggests that the use of TARGIT-IORT would lead to 2,000 fewer deaths from causes other than breast cancer.

Over the last decade, there has been growing support for the use of partial breast irradiation (PBI) instead of whole-breast radiation therapy, and it is arguable that TARGIT-IORT is much better for patients than other methods of PBI (30, 33–35). The TARGIT-A trial cohort comprised a medium-risk population, with a substantial number of patients at a higher risk of relapse: 1,898 (83%) were younger than 70 years, 366 (16%) had tumours >2 cm in size, 443 (20%) patients had grade 3 cancers, 488 (22%) patients had involved nodes, and 426 (19%) had ER- or PgR-negative tumours. Therefore, its results would also be applicable to patients with breast cancer suitable for breast conserving surgery more widely than other methods of PBI (9, 30).

In many countries, patients live a considerable distance from the radiotherapy centre (31, 36, 37) and are more likely to receive a mastectomy than breast conservation (38). Even in the USA as recently as 2015, patients who lived farther away from the radiation facility (> 9.2 miles/19 min away by road) were 36%–44% more likely to receive a mastectomy than breast conservation (38). TARGIT-IORT is a much more convenient option (28, 39). We pointed out that wider availability and applicability of TARGIT-IORT should enable many more women to have the choice of having breast conservation when they would otherwise have a mastectomy because they are not able to have conventional radiotherapy (40–49). TARGIT-IORT also reduces the cost of providing treatment (50–55).

Importantly, TARGIT-IORT lowers the toxicity and reduces deaths from cardiovascular causes and other cancers by a substantial amount (4.4% by 12 years) (30), which has become increasingly important with the rising rates of survival with modern breast cancer treatment. This effect of improving survival appears to be a combination of avoiding the risks due to inadvertent scattered radiation from whole-breast radiotherapy and from a potential abscopal effect of delivery of intraoperative radiotherapy during the surgical excision of the cancer (10).

The strengths of this study are that the data were provided directly by the physicians and staff from each centre and that the response rate of 93% was excellent. In addition, we provide user-friendly interactive links (https://targit.org.uk/travel) for use by clinicians and patients. The obvious weakness is that this paper does not describe data about outcomes, but this is not the intention of this manuscript. Outcome data are best gained from comparative analysis within the prospective TARGIT-A randomised trial (9, 10), as well as data from several centres that have published their own experience of TARGIT-IORT and from prospective registry studies (https://targit.org.uk/publications) (18, 28, 39, 55–65). In addition, as the list of centres using TARGIT-IORT was compiled using personal contacts, we may have missed some centres, underestimating the number of cases. The network of centres using this approach is now been greatly strengthened and will in due course provide the foundation for a unified collection of outcome data.

TARGIT-IORT is now included in several national and international guidelines (66–79) (https://www.targit.org.uk/targit-iort-in-guidelines) for breast cancer treatment. Several of these guidelines specifically recommend using TARGIT-IORT during the COVID-19 pandemic caused by the SARS-CoV-2 virus to give the added advantage of reducing patient exposure to hospital environments and public places.

In this paper, we described the impact of a new treatment proven in a randomised clinical trial over the worldwide breast cancer community. It demonstrates how widely this evidence-based approach has now been adopted and how it has benefitted women with breast cancer around the world.

## Author's note

A pre-print of this paper is available from UCL Discovery http://doi.org/10.14324/000.wp.10121050

## Data availability statement

The raw data supporting the conclusions of this article will be made available by the authors, without undue reservation, and as permitted by individual clinical teams.

## Ethics statement

Ethical review and approval was not required for the study on human participants in accordance with the local legislation and institutional requirements. Written informed consent for participation was not required for this study in accordance with the national legislation and the institutional requirements.

## Author contributions

JV conceived the project and discussed it with UV, MBa, JST, and MBu and wrote the first draft; UV helped in making contacts, collecting data from centres and collating data, and programming for creating the figures and tables; JV, MBa, MBu, JT, DJ, and UV contributed to finalizing the draft. All other authors and contributors/collaborators contributed by treating patients and returning their own data for the compilation and approving the manuscript for submission.

## Conflict of interest

JV has received a research grant from Photoelectron Corp (1996–99) and from Carl Zeiss for supporting data management at the University of Dundee (Dundee, UK, 2004–2008) and has received honorariums. JV and JT received funding from HTA, NIHR, Department of Health and Social Care for some activities related to the TARGIT trials. MBa was briefly on the scientific advisory board of Carl Zeiss and was paid consultancy fees before 2010. Carl Zeiss sponsors some of the travel and accommodation for meetings of the international steering committee and data monitoring committee and when necessary for conferences where a presentation about targeted intraoperative radiotherapy is being made for all authors apart from UV, who has declared no conflict of interest.

The remaining authors declare that the research was conducted in the absence of any commercial or financial relationships that could be construed as a potential conflict of interest.

## Publisher’s note

All claims expressed in this article are solely those of the authors and do not necessarily represent those of their affiliated organizations, or those of the publisher, the editors and the reviewers. Any product that may be evaluated in this article, or claim that may be made by its manufacturer, is not guaranteed or endorsed by the publisher.

## Supplementary material

Supplementary Figure 1 Kaplan-Meier curves showing breast cancer mortality (top left) and non–breast cancer mortality (top right), overall mortality for grade 1 or 2 cancers (bottom left), and grade 3 cancers (bottom left) for TARGIT-IORT v EBRT in the TARGIT-A trial. Figures under titles are hazard ratios (95% confidence intervals) and log rank test P values. EBRT=external beam radiotherapy; TARGIT = targeted intraoperative radiotherapy = TARGIT-IORT (taken from BMJ 2020;370:m2836 https://www.bmj.com/content/370/bmj.m2836.full.pdf and BJC 2021 125, pages380–389 (2021) https://www.nature.com/articles/s41416-021-01440-8.pdf.

Supplementary Figure 2 World map showing countries in which TARGIT-IORT is offered for breast cancer. The shading correlates with the number of centres in each country. For an interactive map see https://targit.org.uk/travel.

Supplementary Figure 3 The number of centres offering TARGIT-IORT increased worldwide from 1998 onward. The graph below includes only those centres from which the date of first case was returned to us.
