# Supplementary material for: Global adoption of single-shot targeted intraoperative radiotherapy (TARGIT-IORT) for breast cancer—better for patients, better for healthcare systems
Source: Front Oncol. 2022 Aug 11;12:786515. doi: 10.3389/fonc.2022.786515 (PMC9406153; doi:10.3389/fonc.2022.786515)

eFigure 1 Kaplan-Meier curves showing breast cancer mortality (top left) and non-breast cancer mortality (top right), overall mortality for grade 1 or 2 cancers (bottom left), and grade 3 cancers (bottom left) for TARGIT-IORT v EBRT in the TARGIT-A trial. Figures under titles are hazard ratios (95% confidence intervals) and log rank test P values. EBRT=external beam radiotherapy; TARGIT = targeted intraoperative radiotherapy = TARGIT-IORT
(taken from BMJ 2020;370:m2836 https://www.bmj.com/content/370/bmj.m2836.full.pdf and BJC 2021 125, pages380–389 (2021) https://www.nature.com/articles/s41416-021-01440-8.pdf

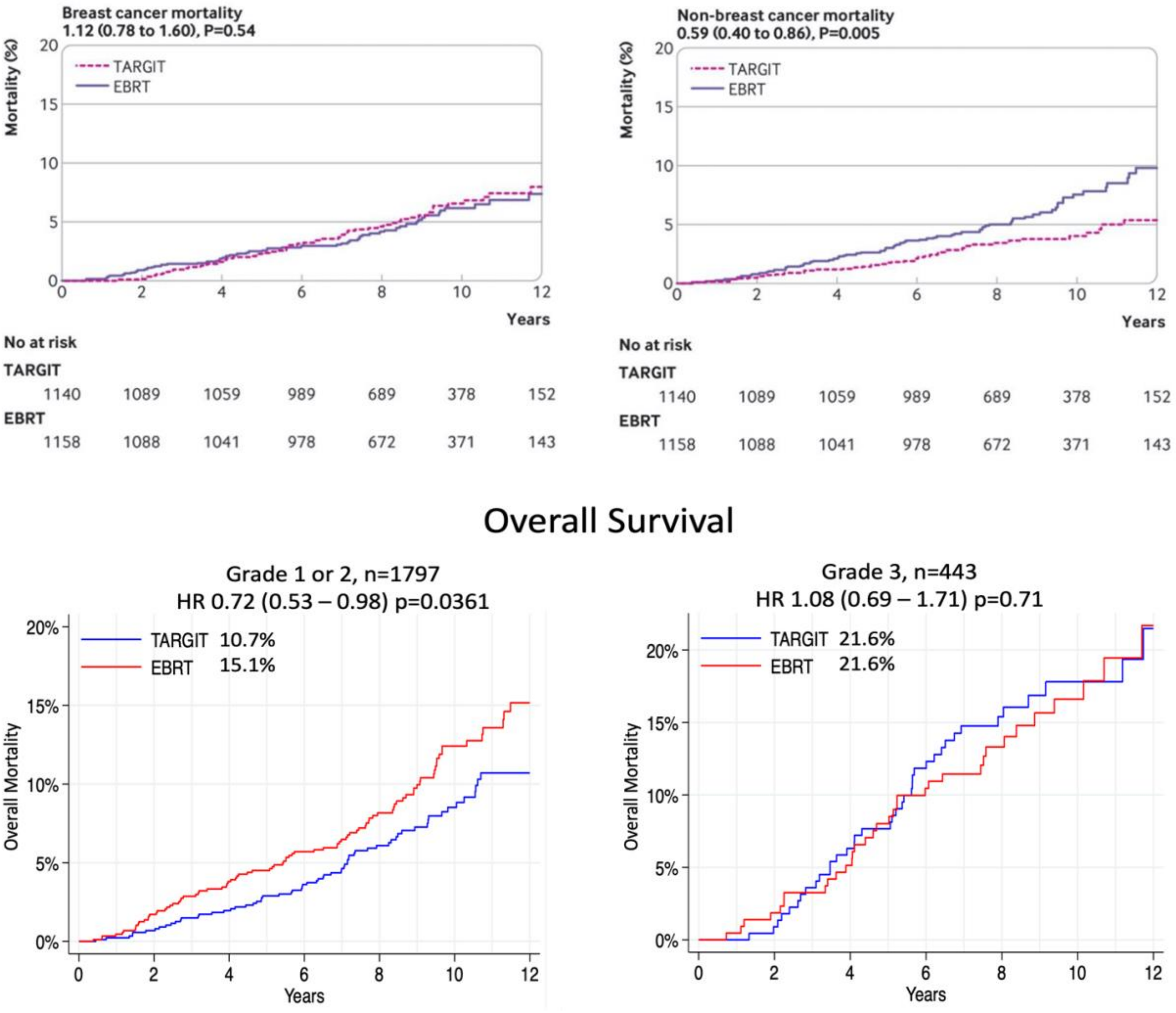

Supplement: Supplementary Figure 1 — Kaplan-Meier curves showing breast cancer mortality (top left) and non–breast cancer mortality (top right), overall mortality for grade 1 or 2 cancers (bottom left), and grade 3 cancers (bottom left) for TARGIT-IORT v EBRT in the TARGIT-A trial. Figures under titles are hazard ratios (95% confidence intervals) and log rank test P values. EBRT=external beam radiotherapy; TARGIT = targeted intraoperative radiotherapy = TARGIT-IORT (taken from BMJ 2020;370:m2836 https://www.bmj.com/content/370/bmj.m2836.full.pdf and BJC 2021 125, pages380–389 (2021) https://www.nature.com/articles/s41416-021-01440-8.pdf. [file Image_1.pdf]
