# Supplementary material for: Global adoption of single-shot targeted intraoperative radiotherapy (TARGIT-IORT) for breast cancer—better for patients, better for healthcare systems
Source: Front Oncol. 2022 Aug 11;12:786515. doi: 10.3389/fonc.2022.786515 (PMC9406153; doi:10.3389/fonc.2022.786515)

eFigure 2 World map showing countries in which TARGIT-IORT is offered for breast cancer. The shading correlates with the number of centres in each country. For an interactive map see https://targit.org.uk/travel

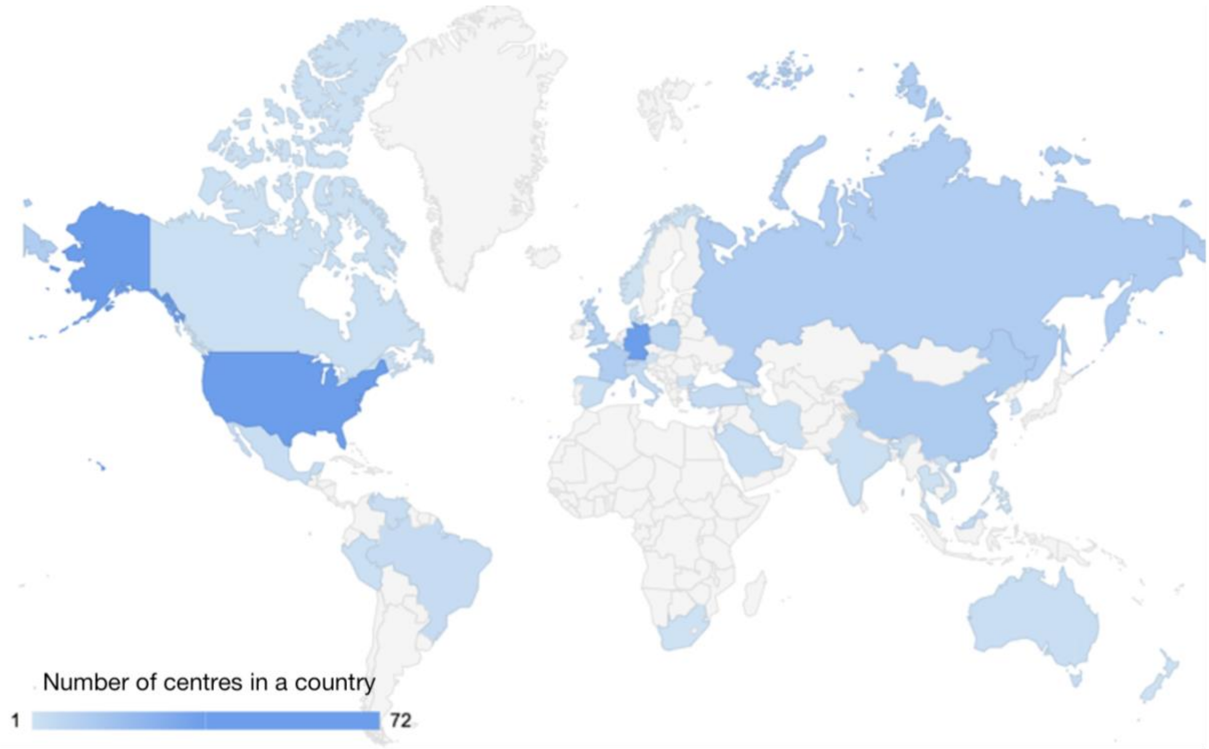

Supplement: Supplementary Figure 2 — World map showing countries in which TARGIT-IORT is offered for breast cancer. The shading correlates with the number of centres in each country. For an interactive map see https://targit.org.uk/travel. [file Image_2.pdf]
