# Supplementary material for: Global adoption of single-shot targeted intraoperative radiotherapy (TARGIT-IORT) for breast cancer—better for patients, better for healthcare systems
Source: Front Oncol. 2022 Aug 11;12:786515. doi: 10.3389/fonc.2022.786515 (PMC9406153; doi:10.3389/fonc.2022.786515)

**eFigure 3** The number of centres offering TARGIT-IORT increased worldwide from 1998 onwards. The graph below includes only those centres from which the date of first case was returned to us.

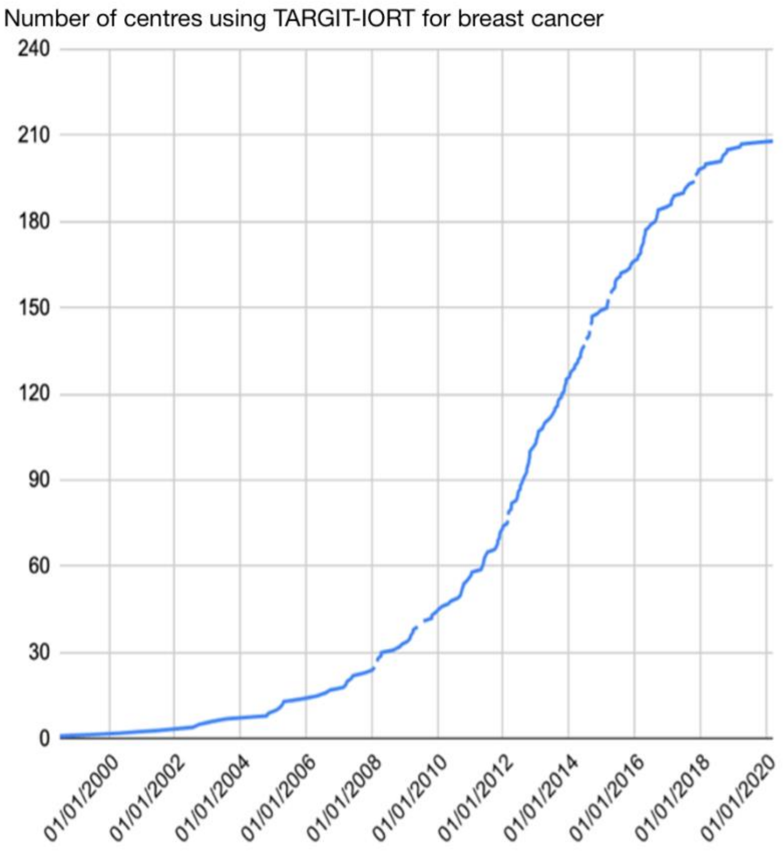

Supplement: Supplementary Figure 3 — The number of centres offering TARGIT-IORT increased worldwide from 1998 onward. The graph below includes only those centres from which the date of first case was returned to us. [file Image_3.pdf]
